# miR-10b is overexpressed in hepatocellular carcinoma and promotes cell proliferation, migration and invasion through RhoC, uPAR and MMPs

**DOI:** 10.1186/s12967-014-0234-x

**Published:** 2014-09-19

**Authors:** Cheng-gong Liao, Ling-min Kong, Ping Zhou, Xiu-li Yang, Jian-guo Huang, He-long Zhang, Ning Lu

**Affiliations:** Department of Oncology, Urumqi General Hospital of Lanzhou Military Command of PLA, No. 359 Youhao North Road, Urumqi, 830000 PR China; Department of Oncology, Tangdu Hospital, Fourth Military Medical University, No. 1 Xinsi Road, Xi’an, 710038 PR China; Cell Engineering Research Centre & Department of Cell Biology, State Key Laboratory of Cancer Biology, Fourth Military Medical University, No. 169 Changle West Road, Xi’an, 710032 PR China

**Keywords:** miR-10b, HCC, invasion, migration, RhoC, uPAR, MMPs

## Abstract

**Background:**

Recently, miR-10b is identified as a miRNA highly expressed in many human cancers, promoting cell migration and invasion. However, the specific function of miR-10b in hepatocellular carcinoma (HCC) is unclear at this point.

**Methods:**

The miR-10b expression levels in 60 paired different TNM Stage HCC tumor tissues compared with adjacent non-tumor (ANT) tissues, normal tissue control (8 benign tumor and 7 normal liver tissues), 3 normal liver and 7 HCC cell lines were measured by real-time quantitative RT-PCR and to evaluate their association with HCC clinicopathologic features. Invasion assay, MTT proliferation assay and wound-healing assay were performed to test the invasion and proliferation of HCC cell after transfection. The effect of miR-10b on HCC *in vivo* was validated by murine xenograft model.

**Results:**

We found that miR-10b expression was increased in human HCC tissues and cell lines compared with normal control, respectively. The expression of miR-10b was correlated with HCC metastatic ability. Overexpression of miR-10b in MHCC-97L cells increased cell motility and invasiveness, whereas inhibition of miR-10b in MHCC-97H cells reduced cell motility and invasiveness *in vitro* and *in vivo*. We also showed that HOXD10 was negatively regulated by miR-10b at the posttranscriptional level, via a specific target site within the 3′UTR by luciferase reporter assay. Furthermore, we found that miR-10b induced HCC cell invasion and migration by modulating the HOXD10 target gene RhoC, uPAR, MMP-2 and MMP-9 expression.

**Conclusions:**

Our results suggested that miR-10b was overexpressed in HCC and promoted HCC cell migration and invasion through the HOXD10/ RhoC/ uPAR/ MMPs pathway which may provide a novel bio-target for HCC therapy.

## Introduction

Hepatocellular carcinoma (HCC) is one of the most common malignancies and leading causes of cancer-related death worldwide, especially in Asian Pacific regions, which has a highly invasive malignant behavior and recurrence rate and remains one of the tumors most refractory to treatment [1].

MicroRNAs (miRNAs), which encode small non-coding RNAs of approximately 22 nucleotides, are now recognized as a very large gene family. Mature miRNAs are thought to regulate the negative expression of a large number of genes carrying target sites within 3′ untranslated regions (UTR) [2]. Recent studies demonstrate that miRNAs may act as activators of tumor metastasis by acting on multiple signaling pathways involved in metastasis [3,4]. Among these miRNAs, miR-10b is first reported to induce breast cancer cell invasion and metastasis [3], which is also found to be associated with tumor invasive potential in pancreatic cancer [5], glioma [6], nasopharyngeal carcinoma [7], esophageal cancer [8] and neurofibromatosis [9]. miR-10b, induced by the pro-metastatic transcription factor TWIST1, proceeds to inhibit translation of mRNA of HOXD10, a transcription factor already known for its roles in cell motility [10], resulting in increased expression of a pro-metastatic gene, RhoC and tumor invasive factors, urokinase-type plasminogen activator receptor (uPAR) [6]. Nowadays, MMPs have also been reported participating in the miR-10b mediated tumor invasion and metastasis [11]. In addition a study detecting the microRNA profiling in hepatocellular tumors compared to benign hepatic tumors and non-tumor liver tissues shown that the expression of miR-10b was increased in HCC [12,13]. But the exact function of miR-10b in HCC is still unknown.

In this study, we first measured miR-10b expression levels in different TNM stage HCC tumor tissues compared with normal liver tissues, and HCC cell lines. Then, we identified the role of miR-10b in HCC cell migration and invasion; furthermore, we also examined the direct binding target of miR-10b. Finally, tumor invasive factors RhoC, uPAR, MMP-2 and MMP-9 were also measured. Thus, our data suggested important roles for miR-10b in HCC development and implicate this miRNA’s potential application as a target for cancer therapies.

## Materials and methods

### HCC and normal liver tissues

Sixty paired tissue specimens of HCC and adjacent non-tumor (ANT) tissues were obtained from Department of Hepatobiliary Surgery in Urumqi General Hospital of Lanzhou Military Command of PLA (Urumqi, China). Eight benign tumor tissues and seven fresh normal liver tissues were also collected as control. Informed consent was obtained from each patient. All the liver samples were divided into 5 groups: non-metastatic HCC tissues (NHCC, patients diagnosed as TNM stage I-II, n = 20), low metastatic HCC tissues (LHCC, patients diagnosed as TNM stage III-IVA with intrahepatic metastasis or regional lymph node metastasis, n = 26), high metastatic HCC tissues (HHCC, patients diagnosed as TNM stage IVB with distance metastasis, n = 14), benign tumor tissues (BT, patients diagnosed as hepatic haemangioma, n = 8), and normal liver tissues (NL, n = 7). All of the tissues were obtained at the time of surgery and immediately stored in liquid nitrogen until use.

### Cell lines and culture conditions

The following cell lines were used in this study: human normal liver cell QZG [14], QSG-7701 [15] and HL-7702 [16]; human hepatocellular carcinoma cell lines: HepG2, BEL-7402, FHCC-98, SMMC-7721, HCC-9724, MHCC-97H (HCC cells with high metastatic potential) and MHCC-97L (HCC cells with low metastatic potential) [17]. All cell lines were purchased from Shanghai Institute for Biological Sciences (Shanghai, China). All cell lines were routinely cultured in RPMI-1640 medium or DMEM medium (Hyclone Laboratories, Logan, UT) supplemented with 10% fetal calf serum (Gibco BRL, Rockville, MD, USA) at 37°C in a humidified atmosphere of 5% CO_2_.

### Real-time quantitative RT-PCR

Total RNA was isolated from tissues and cell lines by using TRIzol reagent (Invitrogen, Carlsbad, CA, USA) according to the manufacturer’s instructions. Reverse transcription was performed using the Prime-Script RT reagent kit (TaKaRa, Otsu, Japan). miR-10b was reverse transcribed by the looped primer, which binds to six nucleotides at the 3′ portion of miR-10b molecules. Reverse transcriptions of RhoC, uPAR, MMP-2 and MMP-9 mRNA were performed according to the manufacturer’s protocol. Real time PCR was performed using SYBR Premix Ex Taq™ II kit (TaKaRa) according to the manufacturer’s protocol on an MX3005P QPCR system (Stratagene, La Jolla, CA, USA). The U6 small nuclear RNA and glyceraldehyde-3-phosphate dehydrogenase (GAPDH) mRNA were used as internal controls for miR-10b and other gene mRNA, respectively. All of the reactions were run in triplicate. The delta-Ct method [18] for relative quantification of gene expression was used to determine miRNAs and mRNA expression levels. The looped RT primer for miR-10 was GTC GTA TCC AGT GCG TGT CGT GGA GTC GGC AAT TGC ACT GGA TAC GAC CAC AAA. The PCR primers for miR-10b were GGA TAC CCT GTA GAA CCG AA and CAG TGC GTG TCG TGG AG T. The primers for U6 were TGG GGT TAT ACA TTG TGA GAG GA and GTG TGC TAC GGA GTT CAG AGG TT. The primers for RhoC mRNA were ATG GCT GCA ATC CGA AAG and GAT CTC AGA GAA TGG GAC AGC. The primers for uPAR mRNA were TCT ATC CGG AGC AGC TGA AAA and CGT GTA GAC GCC TGG CTT GT. The primers for MMP-2 mRNA were CAG GGA ATG AGT ACT GGG TCT ATT and ACT CCA GTT AAA GGC AGC ATC TAC). The primers for MMP-9 mRNA were AAT CTC TTC TAG AGA CTG GGA AGG AG and AGC TGA TTG ACT AAA GTA GCT GGA. The primers for GAPDH mRNA were GGT CGG AGT CAA CGG ATT TG and ATG AGC CCC AGC CTT CTC CAT [8].

### Plasmid construction

For the over-expression of miR-10b, genomic fragment of Homo sapiens miR-10b precursor was amplified by PCR using the primer pairs: 5′-GGC GGA TCC CAA GCC CAT TAG GCT ACC TG-3′ and 5′- CCG GAA TTC TGA GGA GCT TCT GGA GAG GA -3′ [8]. The PCR product was cloned into pcDNA3.1 (Invitrogen) named as pcDNA3.1-miR-10b. 3′UTR segment of HOXD10 was subcloned into the pmirGLO vector (Promega, Madison, WI, USA) immediately downstream of the stop codon of the luciferase gene. The mutant construct of HOXD10 3′UTR was generated using a QuickChange mutagenesis kit (Stratagene, La Jolla, CA, USA) [3]. Wild-type and mutant inserts were confirmed by sequencing.

### Cell transfection

Cultured cells were transfected with miR-10b expression vector, antisense miR-10b (anti-miR-10b), scramble miRNA using Lipofectamine 2000 (Invitrogen) according to the manufacturer’s protocol. miRNA inhibitor was chemically enhanced by 2′O-4′C-methylene (2′-OME) modification. The miR-10b inhibitors and scramble miRNA were designed and synthesized in GenePharma Inc. (Shanghai, China). The sequence was described as before [11].

### Luciferase reporter assay

The cells of 80% confluence in 24-well plates were transiently transfected with firefly luciferase reporter gene constructs and miR-10b expressing plasmid. After 48 h, luciferase activity was measured using a dual luciferase reporter assay system according to the manufacturer’s protocol (Promega), normalized for transfection efficiency by co-transfecting with pRL-TK.

### Cell proliferation assay

Cells were plated in sextuplicate in 96-well plates (2 × 10^3^ per well) in 100 μL complete medium and allowed to attach overnight. 3-(4,5-Dimethyl-2-thiazolyl)-2,5-diphenyl-2H-tetrazolium bromide (MTT) (20 μL at 5 mg/mL; Sigma, St. Louis, MO) was added every 24 h and incubated for 4 h. The supernatant was discarded, the precipitate was dissolved in 200 μL dimethyl sulfoxide (DMSO), and plates were read with a microplate reader at 570 nm [19].

### Wound-healing assay

The wound-healing assay was used to evaluate the tumor cell motility capacity. Briefly, 1 × 10^6^ cells were seeded in six-well plates, cultured overnight, and transfected with miR-10b or miR-10b inhibitor and the respective controls. When the culture had reached nearly 90% confluency, the cell layer was scratched with a sterile plastic tip and then washed with culture medium twice and cultured again for up to 48 h with serum-reduced medium containing 1% FBS. At different time points, photographic images of the plates were acquired under a microscope and the data were summarized based on sextuple assays for each experiment.

### *In vitro* invasion and migration assay

MilliCell (12 mm diameter with 8 μm pores) chambers (Millipore, Bedford, MA, USA) were pre-coated with Matrigel (BD, Bedford, MA, USA) on the upper side. A total of 1 × 10^5^ serum-starved HCC cells were added to the upper compartment in medium supplemented with 0.1% serum, and the chambers were placed into 24-well plates with medium containing 10% serum. After 24 h at 37°C, invaded cells on the lower membrane surface were fixed and stained with 0.1% crystal violet. Invasive activity was quantified by counting nine high-power fields (HPFs, 400×) per chamber. Mean values were obtained from at least three individual chambers for each experimental point per assay. The migration assay is the same with invasion assay excepting no matrigel was used and the permeating time for cells was 12 hours.

### *In vivo* proliferation assay

BALB/c nude mice at 4 to 6 weeks of age were provided by the Laboratory Animal Research Center of Fourth Military Medical University (FMMU), and the animal study was reviewed and approved by Animal Care and Use Committee of FMMU. A total of 5 × 10^6^ MHCC-97L cells stably expressing miR-10b were resuspend in Matrigel (BD, 1 mg/ml) and injected subcutaneously into nude mice. 42 days after injection, the mice were sacrificed. Tumor volume was weekly determined using direct measurement and calculated using the formula length × width^2^/2.

### Western blot

Western blots were performed according to standard protocols using Immobilon-P PVDF membranes (Millipore). For immunoblotting, membranes were incubated with the primary antibody (0.5 μg/mL) for 2 h, followed by a 1 h incubation with HRP conjugated secondary antibody (1:5000). The primary antibodies against HOXD10, RhoC, uPAR, MMP-2, MMP-9 or tubulin were purchased from Santa Cruz Corporation (Santa Cruz, CA, USA). Finally, the blots were washed and the signals were visualized using the ECL plus Kit (Amersham, Buckinghamshire, UK).

### Statistical analysis

Each experiment was performed independently at least twice with similar results; one representative experiment was presented. All statistical analyses were performed using the SPSS 16.0 statistical software package (SPSS, Chicago, IL, USA). The significance of the data was determined using Student’s *t* test. One-way ANOVA was used to compare the miR-10b expression in different groups. All the statistical tests were two-sided, and a *P* value <0.05 was considered significant.

## Results

### miR-10b is over-expressed in HCC tissues and cell lines

**Figure 1:**
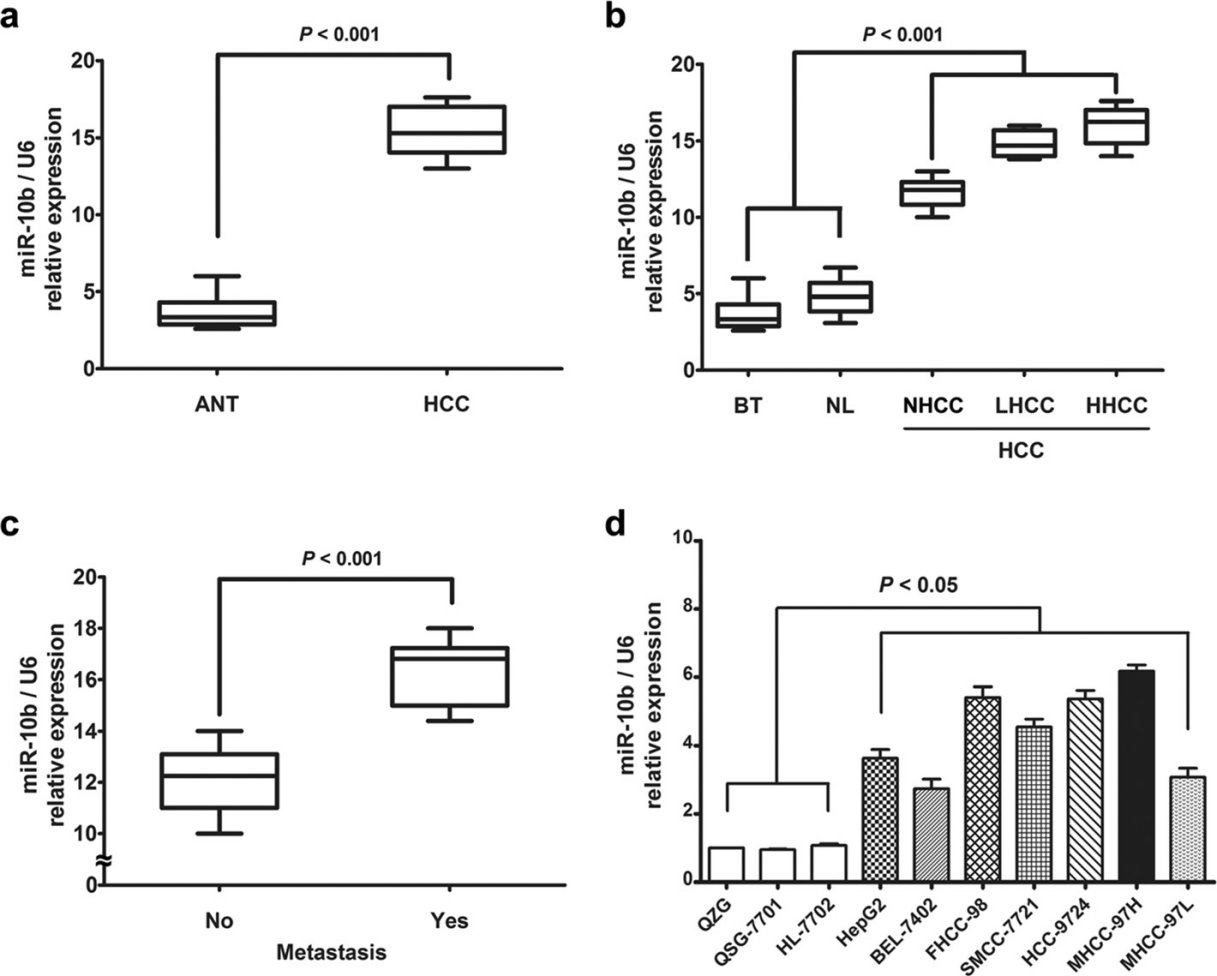
**miR-10b is over-expressed in HCC tissues and cell lines. (a)** The relative levels of miR-10b in sixty paired of HCC samples were measured by real-time quantitative RT–PCR, and the U6 small nuclear RNA was used as an internal control. Student’s *t* test was used to analyze the significant differences between the HCC and ANT tissues. **(b)** The expression of miR-10b in the five tissue groups. Benign tumor tissues and normal liver tissues were counted as control. One-way ANOVA was used to analyze the significant differences among the groups. **(c)** The expression of miR-10b in metastatic HCC (HHCC and LHCC) tissues and non-metastatic HCC (NHCC) tissues. The expression of miR-10b was calculated as the normalized ratio of the expression level of miR-10b in HCC tissues to the expression level of miR-10b in ANT tissues. Student’s *t* test was used to analyze the significant differences. **(d)** The relative levels of miR-10b in the seven HCC and three normal liver cell lines. One-way ANOVA was used to analyze the significant differences.

The expression levels of miR-10b were first evaluated in sixty paired of HCC and ANT tissues by real time RT-PCR. As shown in Figure 1a, miR-10b expression levels were overexpressed in HCC tissues than those in ANT tissues. Then we divided all the liver samples into five groups depending on their pathological diagnosis. When expression levels of miR-10b were compared between subgroups, miR-10b was significantly higher in HCC (NHCC, LHCC and HHCC) groups compared to the normal liver (BT and NL) groups and miR-10b expression was positively correlated with the tumor’s stage (Figure 1b). The expression of miR-10b in metastatic HCC tissues (HHCC and LHCC) was significantly higher than in non-metastatic HCC tissues (NHCC, Figure 1c), which indicated that the miR-10b expression was correlated with the HCC metastatic ability.

We also detected the miR-10b expression in HCC and normal liver cell lines. We performed real-time RT-PCR on a panel of seven HCC and three normal liver cell lines. As shown in Figure 1d, miR-10b expression levels in HCC cell lines were significantly higher than those of normal liver cell lines. miR-10b expression in MHCC-97H, FHCC-98, SMMC-7721 and HCC-9724 cells was relatively higher. In contrast, expression levels of miR-10b in HepG2, BEL-7402 and MHCC-97L cells were relatively lower. The higher expression of miR-10b in HCC cells with high metastatic potential suggested a causal role for miR-10b in the migration and invasion of HCC cells.

### miR-10b promotes HCC cell proliferation, migration and invasion

**Figure 2:**
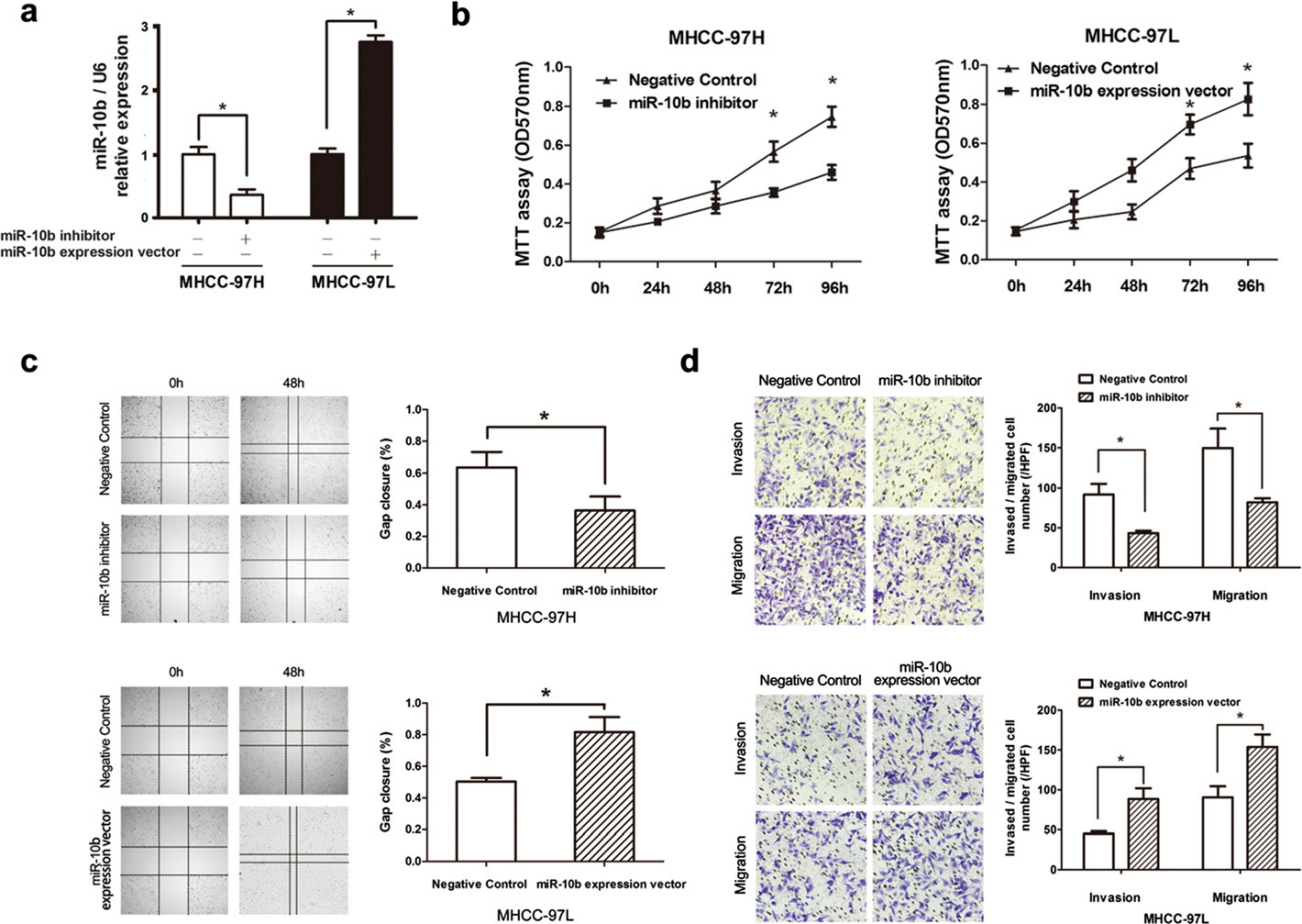
**miR-10b promotes HCC cell migration and invasion. (a)** In MHCC-97L cells transfected with pcDNA3.1-miR-10b and control vector and in MHCC-97H cells transfected with anti-miR-10b, miR-10b expression was measured by real-time quantitative RT-PCR. **(b)** Proliferation of MHCC-97H and MHCC-97L cells transfected with miR-10b inhibitor or miR-22 overexpression vector was measured in the indicated time periods using the MTT assay. **(c)** Wound healing assay of MHCC-97H and MHCC-97L cells transfected with miR-10b inhibitor or miR-22 overexpression vector. **(d)** Migration and invasion potential in both cell lines treated as above were measured by Transwell assays. The results were the means of three independent experiments ± S.D. * *P* < 0.05, Student’s *t* test was used to analyze the significant differences.

To investigate whether miR-10b regulates HCC cell migration and invasion, we selected MHCC-97H cells, which show strong migration and invasion potential, and MHCC-97L cells, which show weak migration and invasion potential, for further study. First, we performed loss-of-function and gain-of-function analysis. miR-10b inhibitor was transfected into MHCC-97H cell to reduce miR-10b expression, while miR-10b expression vector was transfected into MHCC-97L cell to increase miR-10b expression. Then we analyzed miR-10b levels after transfection by real-time RT-PCR and found that miR-10b levels were significantly reduced by the miR-10b inhibitors (decreased about 0.57 fold compared with the control cells, *P* < 0.05, Figure 2a) and elevated by the miR-10b expression vector (increased about 2.75 fold compared with the control in the MHCC-97L cells, *P* < 0.01, Figure 2a).

To examine the role of miR-10b in the proliferation of HCC cells, MTT proliferation assay were performed with MHCC-97H cells transfected with miR-10b inhibitor and MHCC-97L cells transfected with miR-10b expression vector, respectively. The results showed that miR-10b could significantly promoter HCC cell proliferation (*P* < 0.05, Figure 2b). Next, the wound-healing assay showed that MHCC-97H cells with miR-10b inhibitor presented a slower closing of scratch wound, compared with the negative control. There was a quicker closing in MHCC-97L cells with miR-10b overexpression (*P* < 0.05, Figure 2c).

Then we investigated whether miR-10b could enhance HCC cell invasion, transwell assays were performed. The results showed that the inhibition of miR-10b expression led to significant reduction in invasion compared to the control cells (*P* < 0.05, Figure 2d). Then, we utilized miR-10b expression vector to up-regulate the level of miR-10b in MHCC-97L cells, which led to a 2.02 fold increase in invasion (*P* < 0.05, Figure 2d). Next, the effect of miR-10b on the migration of HCC cells was determined by transwell migration assay. In line with the results from the above invasion assays, miR-10b also enhanced the migration of HCC cells (Figure 2d). These observations indicated a positive role for miR-10b in migration and invasion of human HCC cell lines.

**Figure 3:**
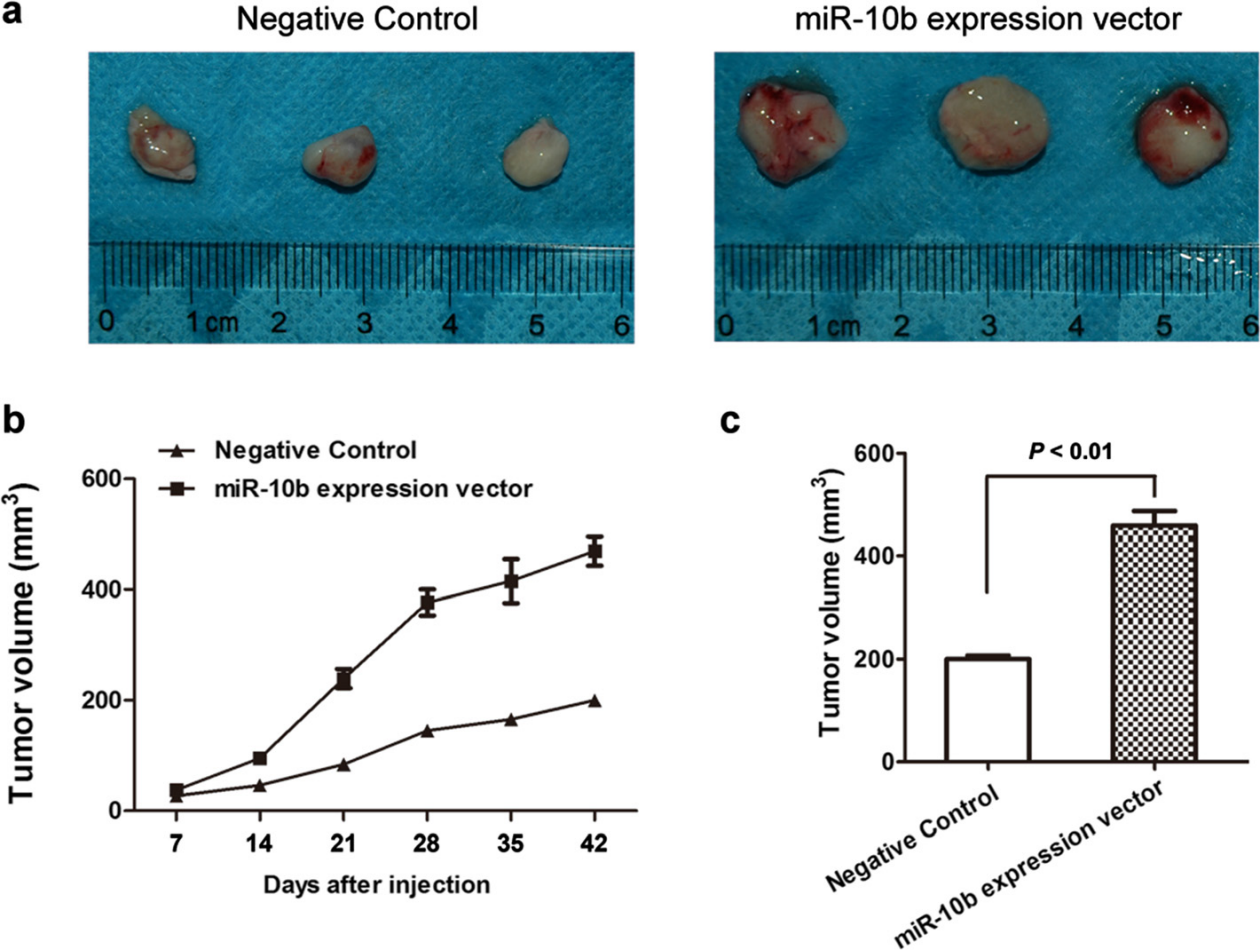
**miR-10b increases HCC growth** ***in vivo*** **. (a)** Representative figures of tumors in negative control and miR-10b overexpression groups. **(b)** Determination of tumor volumes at different time points. **(c)** After the final measure, the mice were sacrificed, and the tumors were excised. Tumor volume was measured and calculated using the formula length × width^2^/2. Student’s *t* test was used to analyze the significant differences.

We next asked whether miR-10b could promote HCC development *in vivo*. Using HCC tumor models, the control mice showed the apparent presence of primary tumor, whereas those injected with miR-10b expression vector increased the volume of tumors during the same observation period (Figure 3). Judging from data between the controls and miR-10b overexpressed groups at the points of the experiment, miR-10b overexpression resulted in a mean increasing in tumor growth.

### HOXD10 is a direct target of miR-10b

**Figure 4:**
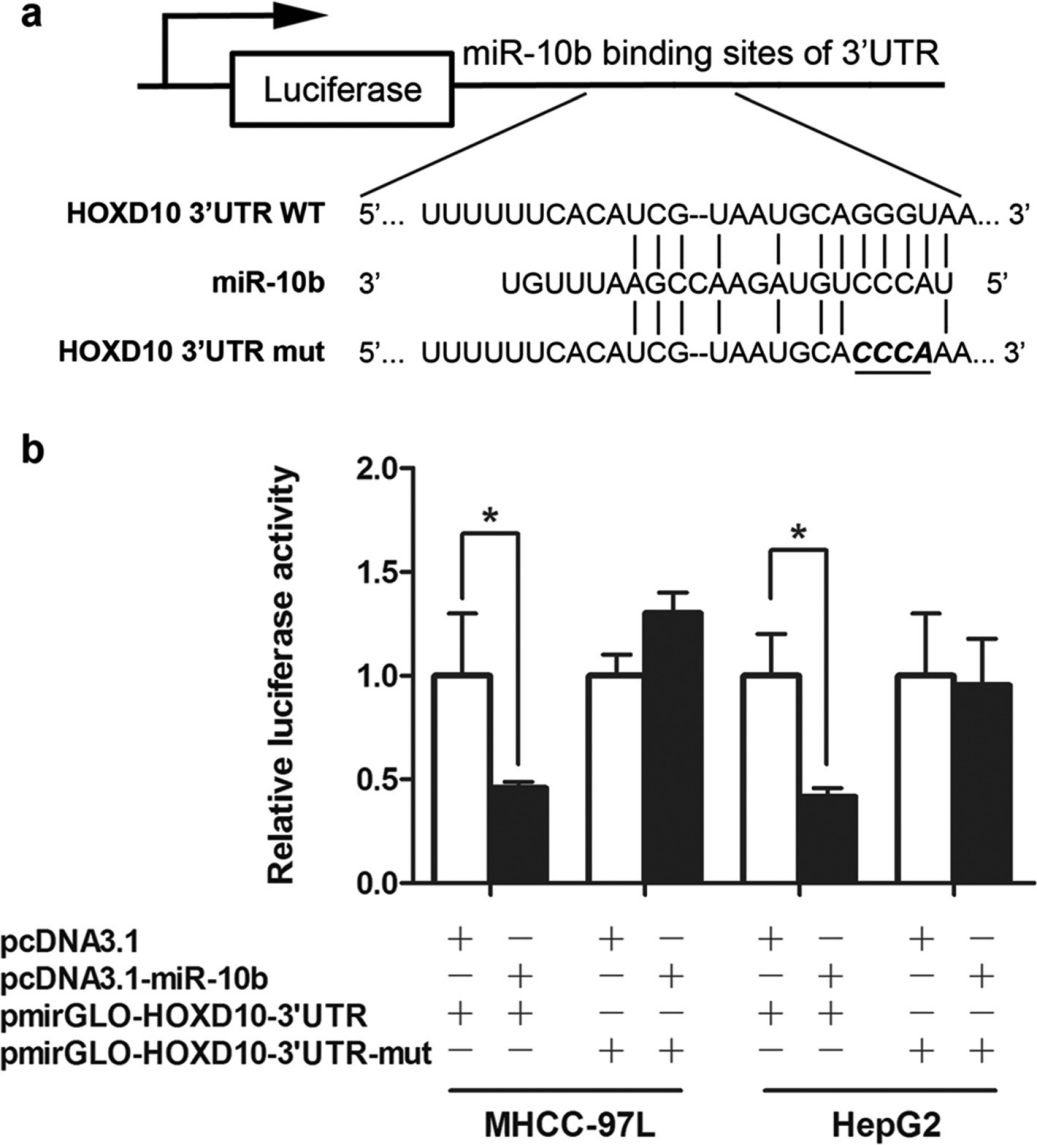
**The HOXD10 3′UTR is a target of miR-10b. (a)** Upper panel, predicted duplex formation between human HOXD10 3′UTR and miR-10b. Lower panel, diagram of the luciferase reporter plasmids: plasmid with the full length wild-type HOXD10 3′UTR (pmirGLO-HOXD10-3′UTR) insert and plasmid with a mutant HOXD10 3′UTR (pmirGLO-HOXD10-3′UTR-mut) which carried a substitution of four nucleotides within the miR-10b binding site. **(b)** Luciferase activity assay demonstrates a direct targeting of the 3′UTR of HOXD10 by miR-10b. MHCC-97L and HepG2 cells were transfected with pcDNA3.1-miR-10b and pmirGLO-HOXD10-3′UTR or pmirGLO-HOXD10-3′UTR-mut. pRL-TK Renilla was used for normalization of transfection efficiency.

HOXD10 has been reported to be regulated by miR-10b in human breast cancer [3]. To verify whether miR-10b directly targeted HOXD10 in HCC cell lines, luciferase reporter assays were carried out. We constructed pmirGLO-HOXD10-3′UTR and pmirGLO-HOXD10-3′UTR-mut with a substitution of four nucleotides within the miR-10b binding site (Figure 4a). Cotransfection of MHCC-97L cells with pmirGLO-HOXD10-3′UTR and pcDNA3.1-miR-10b caused a 0.46 fold decrease in the luciferase activity compared with the negative control (*P* < 0.05). This suppression was rescued by the four-nucleotide substitution in the core binding sites (Figure 4b). The similar effect was also observed in HepG2 cells (0.42 fold decrease compared to the blank control, *P* < 0.05, Figure 4b).

### miR-10b modulates invasion factors RhoC, uPAR and MMPs expression via the target HOXD10 gene

**Figure 5:**
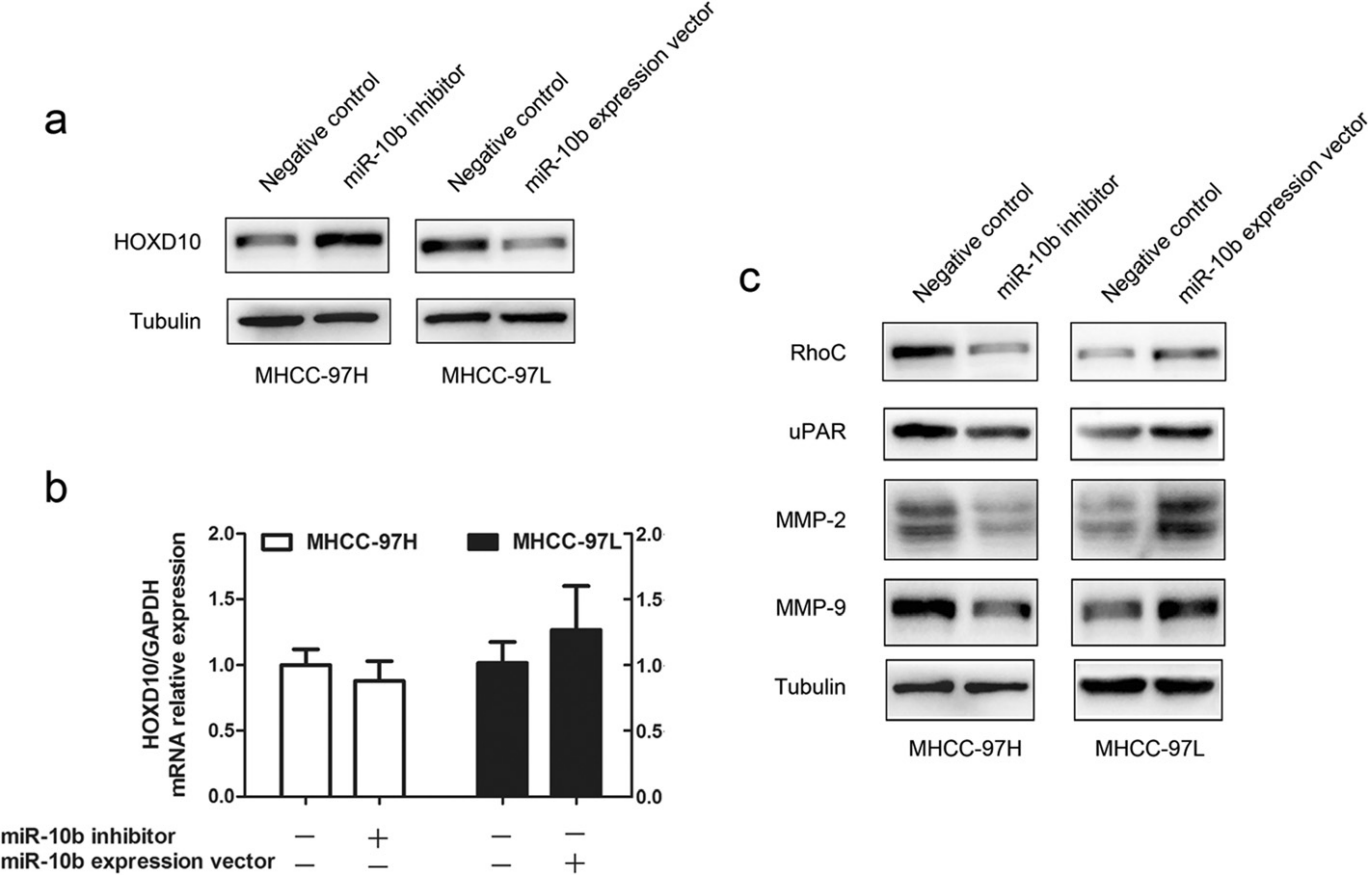
**miR-10b modulates invasion factors RhoC, uPAR and MMPs expression via the target HOXD10 gene. (a)** MHCC-97L cells were transfected with pcDNA3.1-miR-10b and control vector. MHCC-97H cells were transfected with anti-miR-10b and nonsense sequence. Then, HOXD10 protein levels were analyzed by Western blot. **(b)** In parallel, HOXD10 mRNA in the two cell lines treated as above was measured by real time RT-PCR. GAPDH served as internal control. **(c)** The protein expression levels of RhoC, uPAR, MMP-2 and MMP-9 were measured in the two cell lines treated as above was measured by Western blot, respectively.

To test whether miR-10b expression affected endogenous HOXD10 expression, we transfected miR-10b inhibitor and control plasmid into MHCC-97H, and an increase of HOXD10 protein level was observed. Consistent with these results, overexpression of miR-10b in MHCC-97L cells showed a decrease in the HOXD10 protein level (Figure 5a). Real-time RT-PCR showed that overexpression or inhibition of miR-10b had no effect on HOXD10 mRNA level (Figure 5b), which indicated the post-transcriptional regulation of miR-10b on HOXD10 mRNA. Preview studies have shown that miR-10b inhibits the translation of HOXD10 mRNA, thereby affecting the expression of downstream targets of this transcription factor. The potential effectors of HOXD10 include RhoC, uPAR and MMPs, which functions in invasion, migration and metastasis [3,6,11]. To further investigate the signaling pathway, we measured RhoC, uPAR, MMP-2 and MMP-9 protein expression levels after transfection with miR-10b inhibitor in MHCC-97H cells. Expression of all genes was decreased by the miR-10b inhibitor (Figure 5c), which indicated that RhoC, uPAR, MMP-2 and MMP-9 were regulated by miR-10b in HCC cell. Then, we examined the levels of these migration and invasion factors after transfection with miR-10b express vector in MHCC-97L cells. The results showed that all of them were up-regulated by miR-10b over-expression (Figure 5c). These results indicated that miR-10b served as a tumor metastasis factor in HCC cell through the HOXD10/ RhoC/ uPAR/ MMPs pathway.

## Discussion

In our study, we demonstrated that miR-10b expression is increased in human HCC tissues and cell lines compared with matching adjacent non-tumor tissue and normal liver cell lines. The expression of miR-10b correlates with HCC metastatic ability *in vitro* and *in vivo*. It is also demonstrated that HOXD10 is negatively regulated by miR-10b at the posttranscriptional level, via a specific target site within the 3′UTR. miR-10b promotes HCC cell migration and invasion through the HOXD10/ uPAR/ RhoC/ MMPs pathway. Identification of miR-10b as an important regulator of tumor cell migration and invasion emphasizes an essential role of this miRNA in mediating hepatic oncogenesis and tumor behavior.

Increased numbers of reports reveal the aberrant expression of several miRNAs in tumors with a correlation with certain oncogenes or tumor suppressors or in response to chemotherapy or radiation [20-22]. Recently, a growing number of miRNAs have been found to be either activators or suppressors of tumor invasion and metastasis [23-26]. miR-10b was first discovered initiating breast cancer invasion and metastasis [3]. However, the function of miR-10b in HCC cell invasion and metastasis was still unknown. In this study, we investigated the role and the functional target of miR-10b in human hepatocellular carcinoma.

Firstly, we measured miR-10b expression levels in different TNM stage HCC tumor tissues compared with normal liver tissues, and HCC cell lines. Our results showed that miR-10b expression levels were overexpressed in HCC tissues than those in ANT tissues. The expression of miR-10b in metastatic HCC tissues was significantly higher than those in non-metastatic HCC tissues. Furthermore, miR-10b expression in HCC cell with high metastatic potential was statistically significantly higher than that with high metastatic potential HCC cell. These findings indicated that miR-10b is overexpressed in HCC tissues and cells, and might play some role in the invasion and migration of HCC cells.

Also, because miR-10b expression of HCC cell line MHCC-97H was high and MHCC-97L was relatively low in our study, it is interesting to investigate the association between miR-10b expression and the invasive potential or migration levels using these cell lines. We used antisense miR-10b, which could decrease miR-10b expression, and miR-10b expression vector, which could increase miR-10b levels. When transfected with the miR-10b inhibitor, MHCC-97H cells lost their invasion potential while MHCC-97L cells’ invasive ability was enhanced by the miR-10b over-expression. The *in vivo* mice experiments also proved role of miR-10b in HCC proliferation. These results indicated that miR-10b could contribute to the HCC progress through promoting tumor cell proliferation, migration and invasion.

Next we explored the molecular mechanism underlying its function and found that HOXD10 was a direct target of miR-10b in HCC. Luciferase reporter assays showed that miR-10b could bind to the 3′UTR of HOXD10 and down-regulate the luciferase activity. HOXD10, a sequence-specific transcription factor, has been intimately linked with the invasion and metastatic potential of human breast cancer cells [3,27]. HOXD10 expression is lost as a function of invasion and reintroduction of HOXD10 restores non-tumorigenic phenotypes in invasive breast cancer cells [27], suggesting that HOXD10 may play a role as a suppressor of tumor invasive growth. Therefore, HOXD10 is a direct and functional target of miR-10b which may result in an increased expression of a cluster of well characterized pro-metastatic genes.

miR-10b inhibits the translation of mRNA encoding HOXD10, which modulates many genes that promote invasion, migration, extracellular matrix remodeling and tumor progression [10]. So we detected potential target genes of HOXD10, including RhoC, uPAR, MMP-2 and MMP-9. Among these, RhoC has been identified as an especially important player in metastasis [28,29], and its expression correlates with metastatic spread of various types of carcinomas [30-32]. Indeed, we found that miR-10b transduced cells exhibited robust expression of RhoC protein, whereas RhoC expression in the control cells was relatively low. The uPAR has been reported playing a significant role with regard to HCC progression and metastasis [33]. The molecular role of uPAR in cancer progression is well characterized. In addition to its participation in extracellular matrix degradation, uPAR elicits a number of nonproteolytic cellular responses involved in tumor progression and angiogenesis, such as cell migration, adhesion, differentiation, and proliferation [34,35]. One hallmark of metastasis and invasive growth is the transition of tumor cells from an epithelial to a mesenchymal morphology, known as the epithelial–mesenchymal transition (EMT) [36]. MMPs help cancer cells spread by breaking down the extracellular matrix (ECM) and other barriers [37]. MMP-2 and MMP-9 were previously reported to be involved in human tumorigenesis and metastasis [38]. So, we transfected miR-10b inhibitors and found that HOXD10 protein levels were elevated while RhoC, uPAR, MMP-2 and MMP-9 levels were decreased. The transfection of miR-10b expression vector resulted in an opposite effect. These results indicated that miR-10b served as a tumor metastasis factor in HCC cell through the HOXD10/ RhoC/ uPAR/ MMPs pathway.

## Conclusions

The present study provides evidence to support that miR-10b, a microRNA overexpressed in HCC, inhibits the expression of the HOXD10 post-transcriptionally by binding to the 3′UTR of the HOXD10 mRNA, thereby promoting the RhoC/ uPAR/ MMPs induced cell invasion and migration. These findings imply that miR-10b might be a valuable candidate for anticancer therapy.
